# X-pert MTB/RIF^®^ Diagnosis of Twin Infants with Tuberculosis in Da Nang, Viet Nam

**DOI:** 10.3390/jcm6100096

**Published:** 2017-10-17

**Authors:** Phuong Thi Kim Nguyen, Ngu Van Nguyen, Thanh Dinh Phung, Ben Marais

**Affiliations:** 1 Respiratory Department, Da Nang Hospital for Women and Children, Da Nang 550000, Viet Nam; 2 Discipline of Paediatrics and Adolescent Medicine, The Children’s Hospital at Westmead, The University of Sydney, Westmead 2145, NSW, Australia; ben.marais@health.nsw.gov.au; 3 Infectious diseases department, Da Nang Hospital for Women and Children, Da Nang 550000, Viet Nam; bsnguyhnd@gmail.com; 4 Tuberculosis and Lung Diseases Hospital, Da Nang 550000, Viet Nam; thanhbvlbp@gmail.com; 5 Marie Bashir Institute for Infectious Diseases and Biosecurity, The University of Sydney, Westmead 2145, NSW, Australia

**Keywords:** tuberculosis, *Mycobacterium tuberculosis*, infant, child, Gene X-pert

## 1. Summary

4-month-old twins were diagnosed with X-pert MTB/RIF^®^ confirmed tuberculosis (TB). The mother was treated for TB after delivery, but no household contact evaluation was performed or preventive therapy offered. This report illustrates the vulnerability of young children to develop TB, poor implementation of preventive therapy strategies, and the need for meticulous TB exposure assessment in children not responding to pneumonia treatment. 

## 2. Case Report

**Figure 1:**
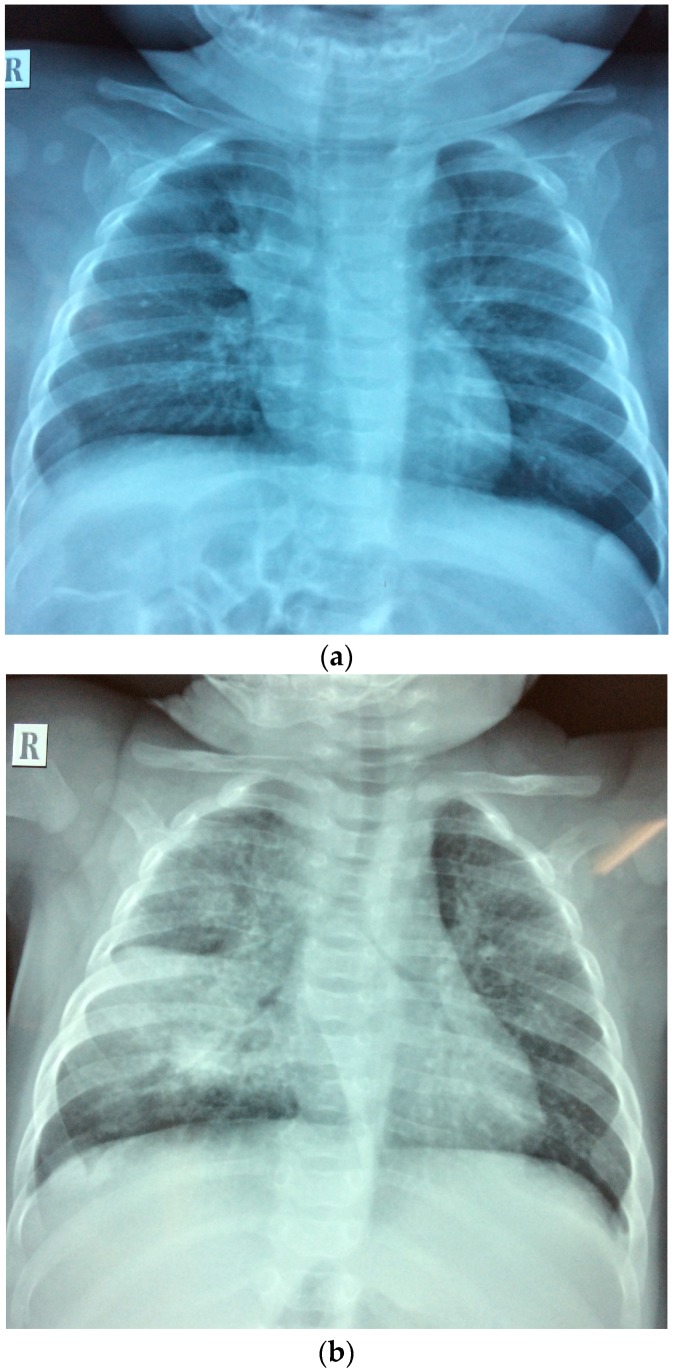
Chest radiographs of (**a**) the index case (twin I) and (**b**) the infant sibling (twin S) at the time of tuberculosis diagnosis.

We admitted a 4-month-old girl (twin I) to the Da Nang Hospital for Women and Children, a provincial referral hospital in central Viet Nam. She presented to our outpatient department with prolonged coughing (around 2 weeks) without any fever or signs of respiratory distress. This persisted despite a 7-day course of oral erythromycin provided by a private paediatrician. She was hospitalized for further diagnostic evaluation. On admission, she weighed 6.2 kg, temperature 37 °C, breathing rate 50/min, heart rate 135 beats/min. On auscultation, her breath sounds were symmetric with a few dispersed crackles and no audible wheezing. Initial investigations, including full blood count and C-reactive protein (CRP), were all in the normal range. However, her chest X-ray (Figure 1a) was suggestive of lymph nodes in the right hilum and mediastinum. A Gene X-pert^®^ test performed on a gastric aspirate (on admission) was positive for *M. tuberculosis* complex, without any *rpo-B* mutations suggestive of rifampicin resistance. The baby was diagnosed with active tuberculosis (TB), and was started on treatment with first-line TB drugs—isoniazid, rifampicin, pyrazinamide and ethambutol—as well as pyridoxine supplementation. The index case was discharged home after 2 weeks in hospital, having demonstrated good drug tolerance and no clinical signs of meningitis or progressive disease. Care will be closely supervised by the local TB clinic with follow-up by doctors at the Tuberculosis and Lung Diseases Hospital in Da Nang, with visits occurring every two weeks during the intensive phase of treatment, and then monthly.

## 3. Relevant Medical History

The index case (twin I) was one of non-identical twins born by C-section at 36 weeks gestation, weighing 2600 grams. She was bottle fed and growing well. She received Bacille Calmette Guerin (BCG) vaccination, but only at 3 months of age, since the parents thought the twins were too premature to be vaccinated at birth. The father smoked around 30 cigarettes/day, and had had a productive cough for many months. He reported no haemoptysis, but did complain of weight loss and malaise. His private doctor diagnosed him with diabetes mellitus, but never evaluated him for tuberculosis. The mother was healthy until delivery, but developed high fever and unilateral chest pain 2 months after giving birth. The mother was diagnosed with TB pleural effusion based on the macroscopic appearance (clear yellow) and lymphocyte predominance of the pleural fluid; smear and culture were negative for *Mycobacterium tuberculosis*. No interferon gamma release assay (IGRA) was performed on the pleural fluid. Given that she was not sputum smear-positive, no household contact tracing was done, and no-one asked about suspicious symptoms in close contacts.

One month later, twin S developed a cough and fever at 3 months of age. She was treated with oral cefuroxime for 10 days, but without any clinical response. She was admitted to Da Nang hospital for Women and Children (the same hospital where twin I was admitted) and diagnosed with pneumonia based on her persistent coughing and chest radiograph abnormalities (Figure 1b). However, she had no signs of acute respiratory distress and all routine investigations were unremarkable. She was given intravenous cefotaxime for 11 days, as well as intravenous gentamycin for 7 days followed by oral erythromycin for 7 days. In the absence of clinical improvement, a high resolution computed tomography (CT) scan of the chest was performed. This revealed multiple nodes in the right hilum and mediastinum. In retrospect, this was visible on the initial chest X-ray, which also suggested possible miliary spread. At this point the mother admitted that she was currently on TB treatment. A Gene X-pert^®^ test performed (2 weeks after admission) on a gastric aspirate was positive for *M. tuberculosis*, without evidence of rifampicin resistance. She had no signs suggestive of meningitis and was started on first-line TB treatment.

**Figure 2:**
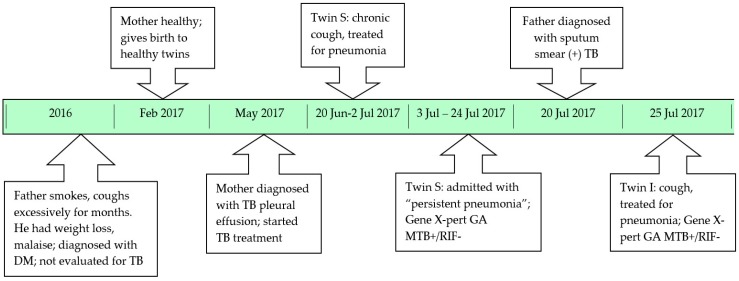
Timeline of the family’s diagnostic journey resulting in twin infants being diagnosed with microbiologically confirmed tuberculosis. TB—tuberculosis; DM—diabetes mellitus; GA—gastric aspirate; MTB—*Mycobacterium tuberculosis*; RIF—Rifampicin.

At the time when twin S was diagnosed with TB the father was screened at a private clinic and found to have sputum smear-positive pulmonary TB. The clinic initiated TB treatment, but did not consider contact screening or the provision of TB preventive therapy to vulnerable household contacts. The diagnosis of twin S with TB also failed to trigger careful evaluation of all household contacts, to perform active case finding, identify the likely source case and provide preventive therapy to those at highest risk of disease development. Twin I (the index case) was brought in for assessment given that she had a persistent cough not responding to first-line antibiotics. A 16-year old sibling was clinically well and not screened. A timeline of the family’s diagnostic journey is provided in Figure 2.

## 4. Discussion

This case illustrates that young children are at very high risk of developing TB following documented TB exposure or infection. The vulnerability of infants and children less than 2 years of age has been demonstrated in descriptive natural history studies conducted in the pre-chemotherapy era [1], and in more recent case series describing nosocomial TB transmission in kangaroo mother care wards [2]. It is rarely appreciated that children with TB, even with fairly advanced disease, usually present without acute respiratory symptoms and with minimal respiratory signs, despite extensive lung and/or intrathoracic lymph node involvement on chest X-ray [3,4]. In fact, this discrepancy between the clinical signs and symptoms observed and the radiological extent of disease is an important clue to consider a TB diagnosis in children. Taking a meticulous history of recent TB exposure is essential, but is often difficult due to time pressures in everyday clinical practice, and because of the considerable stigma associated with TB in certain communities [5]. As in the case presented, family members may be reluctant to volunteer information about TB exposure, and this may be more pronounced in people of higher socio-economic status. Therefore, it is important to ask very specific TB exposure questions in a confidential environment and to acquire as much collateral information as possible in all children with a chronic cough or other suspicious symptoms, including “pneumonia” not responding to first-line treatment [2].

The fact that none of the adult disease episodes triggered an assessment of household contacts was a glaring oversight. Most countries recognize the need for household contact tracing and include the provision of preventive therapy to children less than 5 years of age in their National TB guidelines, but major policy-practice gaps remain [6]. In fact, implementation is mostly absent, with inadequate training, poor resource allocation, and no monitoring system in place to encourage implementation [7]. Although the mother had sputum smear-negative disease, the fact that she had a pleural effusion is highly suggestive of recent primary infection [1] and should have encouraged careful review of recent contact with someone who had symptoms suggestive of TB. The father was diagnosed with sputum smear-positive TB and was probably infectious for a prolonged period, given his chronic and slowly progressive symptoms. As the most likely source case, his diagnosis should have triggered immediate household evaluation, but poor communication between private and public systems often limits the execution of important public health functions. This has been identified as a major obstacle in many Asian countries, contributing to under-reporting of paediatric cases and poor execution of reverse contact tracing, where young children with TB act as a marker of ongoing transmission triggering household contact assessment [8]. Cigarette smoke exposure is an important preventable risk factor for childhood respiratory infections, and high rates of cigarette smoking among men is a concern in Viet Nam and other Asian countries [9]. The fact that the father smoked cigarettes for many years may have masked the onset of TB symptoms, while smoke exposure inside the house would have increased the likelihood of infection among household members.

The infants’ presentation illustrates the importance of considering TB in the differential diagnosis of children not responding to pneumonia treatment [10,11]. It has been demonstrated that TB is an important cause of community-acquired pneumonia not responding to first-line therapy in TB endemic areas [12], but this is rarely emphasized in Integrated Management of Childhood Illness (IMCI) training. Providing nurses and doctors with a systematic approach to diagnosing tuberculosis in resource-limited settings is important [4]. Da Nang Hospital for Women and Children does not have access to IGRA or tuberculin skin testing. These tests do not differentiate *M. tuberculosis* infection from active disease and are not sensitive enough to serve as “rule-out” tests [13]; it essentially provides the same information as careful consideration of recent TB exposure. Any child with persistent, non-remitting symptoms and potential TB exposure should be evaluated for TB with a chest radiograph. Hilar or mediastinal lymphadenopathy, or extensive lung infiltration that seems inconsistent with the child’s non-acute clinical appearance and inflammatory markers, is suggestive of TB [2,6]. The fact that X-pert MTB/RIF^®^ is becoming more widely available should benefit children; good diagnostic utility has been demonstrated in Viet Nam [14]. It is hoped that the much anticipated X-pert Ultra^®^ will have increased sensitivity for detecting pauci-bacillary disease in young children [4].

In conclusion, it is important to emphasize the vulnerability of pregnant women and young children to developing TB [3]. Taking a meticulous TB exposure history is essential in all children with “pneumonia not responding to treatment”, while the diagnosis of an infectious TB case should trigger contact investigation and TB preventive therapy provision to all children less than 5 years of age. This can be achieved even in resource-limited settings by using a pragmatic symptom-based screening approach [15].
